# Honey bee flights near hover under ethanol-exposure show changes in body and wing kinematics

**DOI:** 10.1371/journal.pone.0278916

**Published:** 2022-12-15

**Authors:** Ishriak Ahmed, Charles I. Abramson, Imraan A. Faruque

**Affiliations:** 1 School of Mechanical and Aerospace Engineering, Oklahoma State University, Stillwater, Oklahoma, United States of America; 2 Laboratory of Comparative Psychology and Behavioral Biology, Oklahoma State University, Stillwater, Oklahoma, United States of America; Brown University, UNITED STATES

## Abstract

Flying social insects can provide model systems for in-flight interactions in computationally-constrained aerial robot swarms. The social interactions in flying insects may be chemically modulated and quantified via recent measurement advancements able to simultaneously make precise measurements of insect wing and body motions. This paper presents the first in-flight quantitative measurements of ethanol-exposed honey bee body and wing kinematics in archival literature. Four high-speed cameras (9000 frames/sec) were used to record the wing and body motions of flying insects (*Apis mellifera*) and automated analysis was used to extract 9000 frame/sec measurements of honey bees’ wing and body motions through data association, hull reconstruction, and segmentation. The kinematic changes induced by exposure to incremental ethanol concentrations from 0% to 5% were studied using statistical analysis tools. Analysis considered trial-wise mean and maximum values and gross wingstroke parameters, and tested deviations for statistical significance using Welch’s t-test and Cohen’s d test. The results indicate a decrease in maximal heading and pitch rates of the body, and that roll rate is affected at high concentrations (5%). The wingstroke effects include a stroke frequency decrease and stroke amplitude increase for 2.5% or greater concentrations, gradual stroke inclination angle increase up to 2.5% concentration, and a more planar wingstroke with increasing concentration according to bulk wingstroke analysis. These ethanol-exposure effects provide a basis to separate ethanol exposure and neighbor effects in chemically mediated interaction studies.

## Introduction

Individual insects flying in crowded assemblies perform complex aerial maneuvers by small changes in their wing motions. The complex behaviors and social interactions of honey bees (*Apis mellifera*) make them good candidates for quantifying the individual feedback rules that govern in-flight social interactions between animals. These mathematical rules may be a strong tool informing the design of autonomous aerial robotic swarm implementations on small, computationally-limited robotic platforms. Previous experiments have demonstrated that the degree of honey bee social interaction (and hence these in-flight interactions) may be chemically manipulated through exposure to chemicals such as isopentyl acetate, ethanol, or pheromones such as 9ODA and 9HDA [1].

This study extends prior work on chemical exposure studies in 2D terrestrial locomotion to untethered flight of honey bees by examining the in-flight wing and body kinematics effects of ethanol treatment in honey bees (which have not yet previously been quantified), and by performing statistical analyses on these kinematics relative to unexposed agents. Seventeen motion variables are tracked for each case: 13 body states and 4 gross wingstroke parameters. The analysis approach tests mean and maximum values (computed over each trial) for body states and both trial-wise and bulk wingstroke averaging for wing states. Their statistical significances are determined using Welch’s t-test and Cohen’s d test.

## Previous work

Previous studies support the use of honey bees as a model for chemically-mediated social interactions. Bees engage in a wide range of simple and complex behaviors that include learning and communication. Honey bees foraging on fermenting nectar and fruit may naturally consume ethanol. While honey bees do not have a life stage dependent on alcohol (unlike fruit flies) [2], they readily self-administer high quantities and concentrations of alcohol [3] and demonstrate preferences for specific types of alcoholic drinks [4]. Honey bees have shown preference to ethanol containing solutions over sucrose-only solutions even if they find the test aversive [5]. Bees and humans have been recorded exhibiting parallel aggression, locomotor, and learning changes following ethanol consumption [6, 7]. Ethanol reduces the sting extension response threshold [7] and increases the number of stings [6]. High levels of exposure negatively impact passive avoidance learning [8].

Locomotor activity decreases are dose-dependent [9], with small quantities inducing erratic movements [10] and high EtOH doses inducing decreases in both bee flight and walking activity [3, 11]. Free flight foraging behaviors suggest the species can build ethanol tolerance [8, 12] showing adaptive reactions to ethanol exposure [13]. They also show withdrawal symptoms after discontinuation of ethanol consumption [14]. Amounts of alcohol dehydrogenase and levels of resistance to alcohol intoxication vary by caste [15].

Ethanol dose-dependent learning impairments have also been recorded in honey bees [9, 16, 17], even in learning tasks as simple as association between an odor (conditioned stimulus) and a sucrose reward (unconditioned stimulus) in proboscis extension response (PER) experiments e.g. [9].

The previous work indicates that general honey bee behavior is changed under ethanol influence and suggests their flight behavior may potentially be impacted as well. However, a review of archival literature shows that digitized recordings of in-flight wing and body motions for ethanol-exposed honey bees have not previously been reported. In this study, high speed visual tracking is used to measure body and wing motion states in flight after consumption of ethanol concentrations from 0% to 5%. Statistical tests (Welch’s t-test, Cohen’s d effect size) are then applied to those measurements to reveal the effects of ethanol consumption.

## Methods and approach

### Experimental procedure

#### Chemically-exposed honey bee preparation

Foragers exiting a research hive were captured and anesthetized via storage below 0° C for 3 minutes and restrained in a harness made from a modified microcentrifuge tube. The insects were fed sucrose solution until no PER was present and were allowed to rest for approximately 24 hours at 22° C. This preparation ensured a consistent metabolic state at the beginning of experiments [18] and minimized STRANGE effects [19]. The insects were then fed sucrose-based solutions with varying ethanol concentrations [9]. Feeding was initiated by touching the antennae of bees to initiate a proboscis response (individuals without a PER upon antenna stimulation were eliminated from the study). Subjects were allowed to drink from a microcentrifuge cap containing 20 microlitres of solution until satiated. Feeding was stopped when there was no more PER (in approximately 80 seconds), at which point approximately 0–6*μ*L remained. This treatment process was consistent with [9], where each insect consumed approximately 15 microliters of solution. Then each insect was kept for 15 minutes before adding them to the flight test chamber. Each insect was removed from the test chamber less than 40 minutes after introduction to ensure the flight is recorded only under chemical influence.

#### High speed kinematics measurement

**Fig 1:**
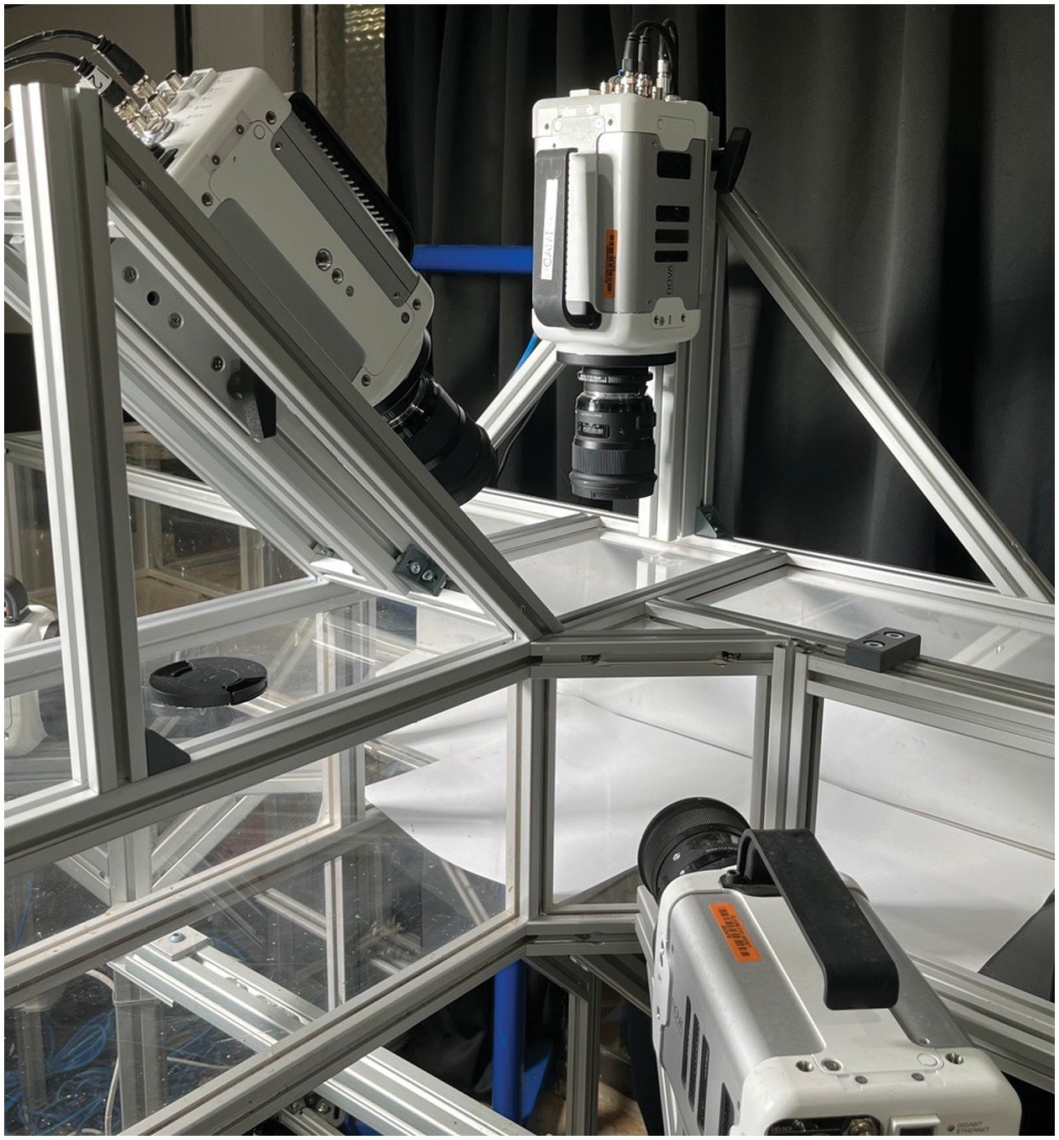
Tunnel setup attached to a beehive with camera setup to film the intersection.

**Fig 2:**
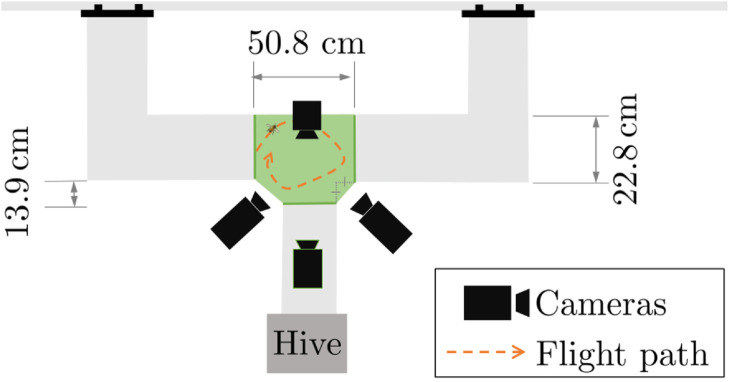
The intersection region is confined (shown in green) and served as the test volume for control and ethanol exposed insect flight.

A transparent T-shaped tunnel was attached to an *Apis mellifera* hive entrance with the two remaining exits exiting to outdoor space as seen in Fig 1. Multiple partitions were added to confine the insect in the test area as seen in Fig 2. Four Photron high speed cameras filmed the T-joint intersection at 9000 Hz. The intersection was isolated with partitions in order to work as a confined 38309.67 cm^3^ test volume, with a 14349.66 cm^3^ simultaneous capture volume. Recording was initiated manually when the insects started flying in the visible volume and ended when they left the volume covered by at least 3 cameras.

#### Digitizing tool

Recorded insect flight trajectories were digitized using a high speed visual insect swarm tracker (Hi-VISTA) [20] implemented in MATLAB which can provide high-resolution tracking of multiple insects using a multiple camera system. The Hi-VISTA tracker takes synchronized frames from different cameras, identifies and removes background to recover multiple insect “blobs” which are then associated in different views. These insect targets are reconstructed through voxel carving by checking consistency in views with the aid of camera projection matrices. Using the reconstructed insect visual hull, Hi-VISTA then segments each insect into wing and body and applies principal component analysis to vector geometry to determine their poses.

### Analysis

#### Body parameters considered

For a time history over [0, *T*_*r*_], where *T*_*r*_ is the time length recorded, time *t* was discretized as *t*_*i*_, *i* = 1, 2, 3…, *n* at a constant sample frequency, and the mean value of a variable *h*(*t*) measured in the flight sequence was calculated as

$$\begin{array}{c} \bar{h}≔\frac{1}{n}\sum_{i=1}^{n}h\left(t_{i}\right),\;t_{i}\in \left[0,T_{r}\right] \end{array}$$ (1)

and the maximum value is defined as

$$\begin{array}{c} h_{\text{max}}≔\underset{t\in [0,T_{r}]}{\text{max}}\left[h\left(t\right)\right]. \end{array}$$ (2)

Body parameters in each flight sequence were characterized by 13 scalar values as shown in Table 1. We define the set of these scalars as *B*. Each of these variables is measured from the stability axes of the insect. For each *s* ∈ *B* we consider trial-wise mean and standard deviation.

**Table 1:** Characterizing body *B* and wing *W* variables in flight sequence.

Set	Notation	Description
*B*	$\bar{\theta_{b}}$	Mean body pitch angle
	$\bar{\left|u\right|}$	Mean absolute forward speed
	$\bar{\left|v\right|}$	Mean absolute sideways speed
	$\bar{\left|w\right|}$	Mean absolute heave speed
	$\bar{\left|p\right|}$	Mean absolute roll rate
	$\bar{\left|q\right|}$	Mean absolute pitch rate
	$\bar{\left|r\right|}$	Mean absolute yaw rate
	|*u*|_max_	Maximum absolute forward speed
	|*v*|_max_	Maximum absolute sideways speed
	|*w*|_max_	Maximum absolute heave speed
	|*p*|_max_	Maximum absolute roll rate
	|*q*|_max_	Maximum absolute pitch rate
	|*r*|_max_	Maximum absolute yaw rate
*W*	*f*	Peak wingstroke frequency
	Φ	Peak wingstroke amplitude
	*β*	Stroke plane angle
	*δ*	Dorsal bias

The trial-wise mean value of a body variable *s* was defined as

$$\begin{array}{c} \mu \left(s\right)≔\frac{1}{n}\sum_{i=1}^{n}s_{i}. \end{array}$$ (3)

where *n* is the number of flight sequences recorded in the respective category (0%, 1%, 2.5%, 5%). The trial-wise standard deviation of a body variable was defined as

$$\begin{array}{c} \sigma \left(s\right)≔{\left(\frac{1}{n}\sum_{i=1}^{n}{\left(s_{i}-\mu \left(s\right)\right)}^{2}\right)}^{1/2}. \end{array}$$ (4)

#### Wing parameters considered

The insect wingstrokes were analyzed as a set *W* comprised of 4 scalar variables.

**Fig 3:**
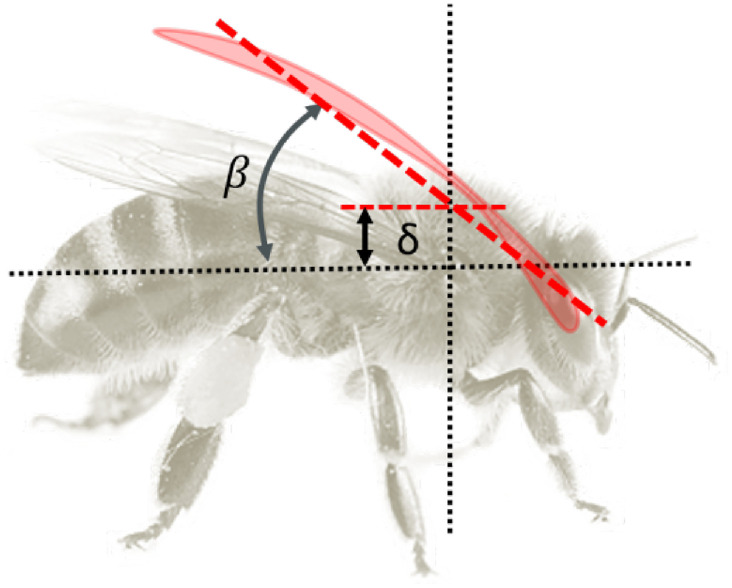
Definition of wing variables. Stroke plane angle *β* and dorsal bias *δ* are determined relative to planar wingtip motion.

The wing stroke, elevation, and pitch angles are represented as 3–1-2 Euler angles (*ϕ*, *ψ*, *α*). Gross stroke frequency was determined by peak to peak time difference *T*_*p*_ = 1/*f* in *ϕ*(*t*) of left wing. *ϕ* and *ψ* time histories are then resampled to have a fixed number of discrete data points *N* over each wingstroke. For each wingstroke, the stroke plane angle *β* and dorsal bias *δ* is determined as seen in Fig 3 by fitting

$$\begin{array}{c} \psi (k)=-\phi (k)\text{tan}\beta +\delta ,\;k\in [1,N]. \end{array}$$ (5)

*β* can be used to compute the planar motion of wing as in [21, 22] by

$$\begin{array}{c} \gamma (k)=\phi (k)\text{cos}\beta -\psi (k)\text{sin}\beta \;k\in [1,N]. \end{array}$$ (6)

The stroke amplitude Φ for the wingstroke can be determined from the peak frequency of the Fourier transform of *γ*. Both wing motions were considered while determining *β*, *δ*, Φ by concatenating the datapoints.

The wingstroke motion characteristics are analyzed by considering bulk averaging (a mean across all wingstrokes at a given concentration), or by first computing a mean across the wingstrokes in each trial before the concentration analysis. The analysis was done as follows:

#### Bulk wingstroke wise analysis

For bulk wingstroke data analysis, mean and standard deviation of these variables *s* ∈ *W* are determined as

$$\begin{array}{c} \mu \left(s\right)≔\frac{1}{2M}\sum_{i=1}^{2M}s_{i}, \end{array}$$ (7)

$$\begin{array}{c} \sigma \left(s\right)≔{\left(\frac{1}{2M}\sum_{i=1}^{2M}{\left(s_{i}-\mu \left(s\right)\right)}^{2}\right)}^{1/2}, \end{array}$$ (8)

where *M* is the number of total wingstrokes recorded in the respective category (0%, 1%, 2.5%, 5%).

#### Trial-wise analysis

A bulk averaging approach gives stronger weight to longer trials. Trial-averaging prior to concentration analysis was used to reduce this effect. In this approach, the mean and standard deviation of variables *s* ∈ *W* are determined by first computing a trial-wise average *μ*_*j*_ over the recorded *m*_*j*_ wingstrokes. This trial has a mean parameter

$$\begin{array}{c} \mu_{j}\left(s\right)≔\frac{1}{2m_{j}}\sum_{i=1}^{2m_{j}}s_{i}. \end{array}$$ (9)

The concentration’s mean and standard deviation may then be found as

$$\begin{array}{c} \mu \left(s\right)≔\frac{1}{n}\sum_{j=1}^{n}\mu_{j}\left(s\right) \end{array}$$ (10)

and

$$\begin{array}{c} \sigma \left(s\right)≔{\left(\frac{1}{n}\sum_{j=1}^{n}{\left(\mu_{j}\left(s\right)-\mu \left(s\right)\right)}^{2}\right)}^{1/2}, \end{array}$$ (11)

where *n* is the number of total insects recorded at the respective concentration (0%, 1%, 2.5%, 5%).

#### Statistical analysis tools

In order to identify variables where data showed statistical differences, binary statistical analysis was applied by dividing the data into groups (*G*_1_: 0%, *G*_2_: 1%, *G*_3_: 2.5%, *G*_4_: 5%, *G*_5_: 1, 2.5, 5%).

The statistical tools applied to this dataset were Welch’s t-test and Cohen’s d test. Welch’s t-test tests the null hypothesis that two populations have equal means for some variable. This hypothesis was tested for each *s* ∈ *S* and *s* ∈ *W* where the null hypothesis is

$$\mu_{G_{1}}\left(s\right)=\mu_{G_{i}}\left(s\right),\;i=2,3,4,5$$

Welch’s t-test does not assume equal variance and is helpful when sample sizes are not equal. *p*-values are used to indicate the probability of the null-hypothesis being true. Cohen’s d test quantifies effect size by *μ*(*s*) deviation in terms of pooled standard deviation.

## Results and discussion

In this experiment, we recorded flights of bees exposed with 20% sucrose and four ethyl alcohol concentrations by volume: 0% (Control), 1%, and 2.5% and 5%. 33 flight sequences were recorded with 33 insects (9 in 0%, 8 in 1%, 8 in 2.5%, 8 in 5% respectively). Overall, the 0%, 1%, 2.5%, and 5% trials contain 1499, 1367, 888, and 1036 wingstrokes, respectively.

**Fig 4:**
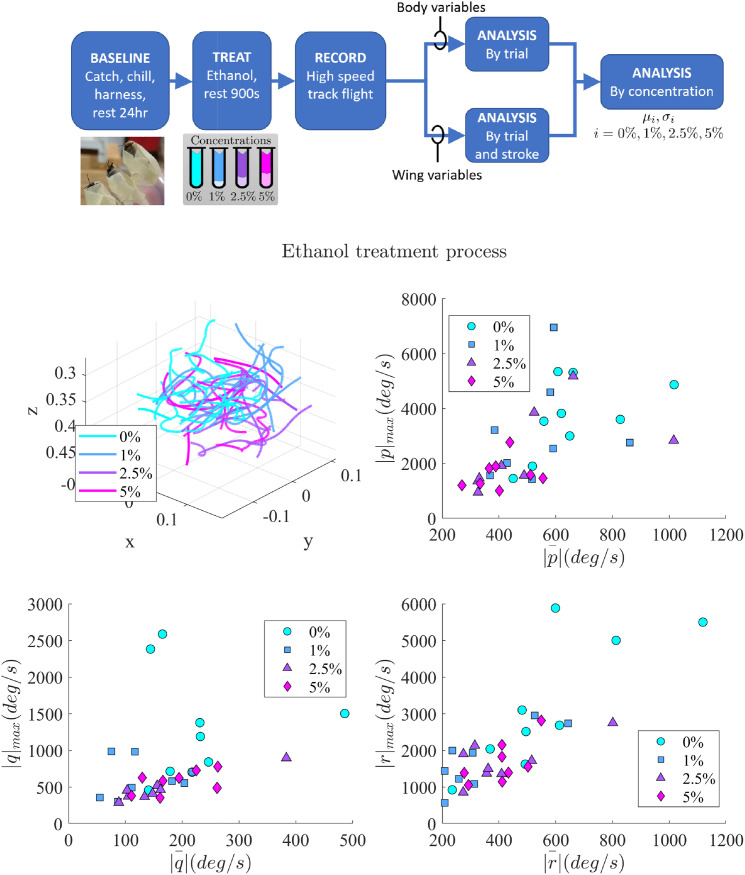
Experimental procedure and body variable characteristics. A flowchart shows the experimental process. 3D flight trajectories show the untethered flights analyzed. Maximum vs. average body rotation rates for roll (*p*), pitch (*q*), and heading (*r*). Maximal heading and pitch rate both decrease with ethanol exposure. Roll rate is affected in 5% concentration.

**Fig 5:**
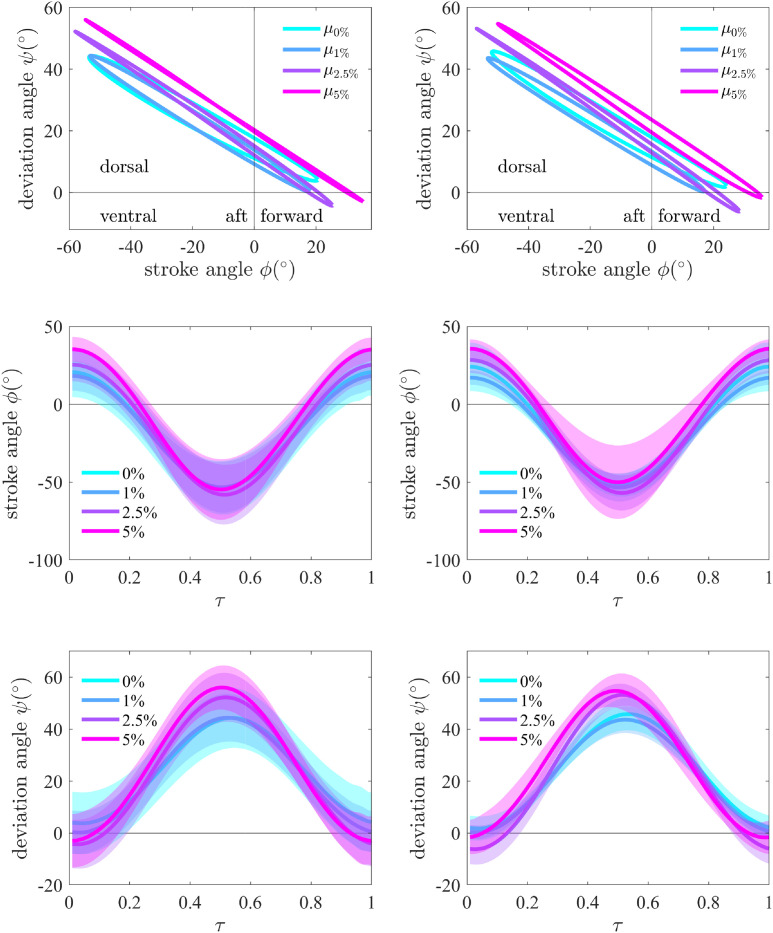
Wing kinematics: Bulk (left column) and trial-wise (right column). Stroke vs deviation angle of each concentration’s mean wingstroke pattern (top row) shows the increasingly planar strokes with concentration in bulk analysis, while the individual analysis shows deviation from the trend in 5% concentration. Wing stroke and deviation angle stroke histories (bottom 2 rows, *τ* denotes non-dimensional time) are shown as for each concentration’s mean stroke *μ* ± its standard deviation *σ*. Stroke histories illustrate how the ethanol-related changes exceed standard deviations.

The overall data is summarized in Tables 2 and 3, and the raw data characteristics of the affected variables are presented in Figs 4 and 5. The following sections detail ethanol-related reductions in behavior, maximum heading and pitch rates, decrease in wing frequency and loop size and increase in amplitude and inclination angle.

**Table 2:** Mean *μ*_*i*_ and standard deviation *σ*_*i*_ of *i* = [0%, 1%, 2.5%, 5%] concentration datasets. Asterisks indicate significant p-values (***<0.001, **<0.01, * < 0.05).

Variable	0% (control)		1%		2.5%		5%	
	*μ*_0%_	*σ*_0%_	*μ*_1%_	*σ*_1%_	*μ*_2.5%_	*σ*_2.5%_	*μ*_5%_	*σ*_5%_
$\bar{\theta_{b}}$ (deg)	38.33	8.43	30.78	10.47	40.18	15.79	44.55	8.51
$\bar{\left|u\right|}$ (m/s)	0.30	0.08	0.32	0.08	0.28	0.10	0.34	0.17
$\bar{\left|v\right|}$ (m/s)	0.19	0.05	0.17	0.07	0.14	0.09	0.21	0.10
$\bar{\left|w\right|}$ (m/s)	0.12	0.03	0.17	0.09	0.16	0.13	0.16	0.06
$\bar{\left|p\right|}$ (deg/s)	656.57	171.16	540.40	158.75	510.14	235.92	408.17**	92.95
$\bar{\left|q\right|}$ (deg/s)	226.93	104.92	131.17*	61.97	159.95	94.17	188.87	57.16
$\bar{\left|r\right|}$ (deg/s)	579.67	258.26	337.52*	160.85	413.47	175.33	410.98	92.62
|*u*|_max_ (m/s)	0.56	0.09	0.47	0.10	0.41**	0.10	0.54	0.16
|*v*|_max_ (m/s)	0.50	0.15	0.37*	0.10	0.35*	0.12	0.42	0.18
|*w*|_max_ (m/s)	0.37	0.11	0.39	0.17	0.29	0.12	0.37	0.10
|*p*|_max_ (deg/s)	3648.32	1391.59	3131.99	1847.50	2388.81	1465.98	1623.18**	551.47
|*q*|_max_ (deg/s)	1306.49	750.40	620.02*	258.81	472.10*	185.23	570.74*	152.61
|*r*|_max_ (deg/s)	3253.09	1784.58	1741.28*	821.30	1699.60*	574.15	1665.31*	584.09

**Table 3:** Mean *μ*_*i*_ and standard deviation *σ*_*i*_ of *i* = [0%, 1%, 2.5%, 5%] concentration datasets for wing variables for trial-wise and bulk wingstrokewise analysis. Asterisks indicate significant p-values (***<0.001, **<0.01, * < 0.05).

Analysis Type	Variable	0% (control)		1%		2.5%		5%	
		*μ*_0%_	*σ*_0%_	*μ*_1%_	*σ*_1%_	*μ*_2.5%_	*σ*_2.5%_	*μ*_5%_	*σ*_5%_
Bulk wingstroke-wise Eqs (7) and (8)	*f* (Hz)	239.25	15.65	254.18***	12.61	229.88***	14.26	223.17***	14.81
	Φ (deg)	43.86	8.01	42.68***	9.40	51.38***	7.86	55.08***	7.01
	*β* (deg)	27.41	9.60	31.40***	5.91	33.99***	7.35	33.70***	6.80
	*δ* (deg)	15.81	7.72	11.96***	4.73	14.30***	6.77	21.10***	6.80
Trial-wise Eqs (10) and (11)	*f* (Hz)	233.33	15.06	253.01**	10.22	226.13	11.61	221.80	13.23
	Φ (deg)	46.32	7.00	41.95	6.58	53.04*	5.80	54.73*	7.39
	*β* (deg)	29.23	5.53	31.37	2.12	34.79*	3.62	34.53	5.17
	*δ* (deg)	16.14	3.42	11.48**	2.17	14.85	4.08	23.55*	8.33

### Insect behavioral context

**Fig 6:**
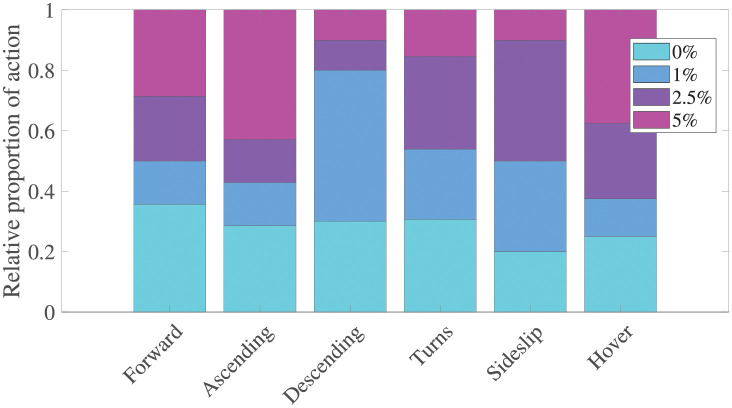
Behavioral repertoire distribution by ethanol concentration. Behavioural diversity was estimated via proportional analysis on manual labels. The results indicate a dominance of turning flight, where behaviors were consistent across concentrations, and that 1% and 5% trials had higher incidences of descending and ascending flight, respectively.

A trial’s advance ratio $J=u/(|\dot{\phi }|R)$ with *R* being wing length quantifies forward speed relative to the maximum wingtip speed. Advance ratio remained below 0.1 across the dataset, which is consistent with rotorcraft and biology definitions of near hover conditions [23–25]. Angular rates were not necessarily small, and the dataset included a diversity of behaviors, as illustrated by the generally circuitous maneuvering and high body rates as seen in Fig 4. Previous work has focused on quantifying flight behaviors via propensity, phototaxis, number of take-offs, and duration, [26] in part due to flight being composed of short bursts [27]. These studies show that flight behavior can differ depending on the flight assay and underlying apparatus. Our focus on in-flight kinematic characteristics requires an awareness of the in-flight behavior repertoire as well. Each trial was manually classified using six labels (forward motion, ascending, descending, turning, sideslips, and pure hover) to estimate the diversity of motion. The dataset shows all trials were biased towards turning behaviors (41% of applied labels), with a smaller representation across the other labels: 15% forward flight, 15% ascending, 11% descending, 11% sideslip, and 8% hovering. The proportionality analysis in Fig 6 indicates that the dataset shows no clear trend across the dominant turning sequences, while 1% and 5% exposed trials had higher incidences of descending and ascending flight, respectively.

### Body variable characteristics

#### Pitch rates: $\bar{\left|q\right|},{\left|q\right|}_{\text{max}}$

Mean absolute pitch rates decreased in all percentages but the decrease is significant only in the 1% case (*p* = 0.03, *d* = −1.09). Maximum absolute pitch rate |*q*|_max_ is significantly reduced in all comparisons (*G*_2_, *G*_3_, *G*_4_, *G*_5_) with (*d* < −0.8). Overall, the changes in maximum pitch rates between control and exposed group (*G*_5_) have a large Cohen-d effect size with *p* = 0.01, *d* = −1.79. A number of previous analyses indicate that airframe pitch modes are often unstable without neural feedback [28–30]. This shift could signify that the pitch rate control mechanisms may have been affected and the unexposed insects’ flight envelopes include more aggressive motions, which is supported by the behavioral analysis’ indication of more diverse behaviors in the control group.

#### Heading rate $\bar{\left|r\right|},{\left|r\right|}_{\text{max}}$

The overall decrease in mean absolute heading rates is significant only in the 1% case (*p* = 0.03, *d* = −1.09). Maximum absolute heading rate |*r*|_max_ is significantly reduced in all comparisons (*G*_2_, *G*_3_, *G*_4_, *G*_5_), with a large Cohen-d effect sizes (*d* < −0.8). This study’s dominance of turning behaviors, combined with the relatively consistent proportion of turning labels across concentrations, reinforces the link between the observed heading rate reduction and ethanol concentration. Previous work has indicated that “flapping counter-torque” provides passive stabilization through aerodynamic damping on this axis [31, 32], suggesting the reduction in maximum heading rate may have different interactions with the underlying airframe relative to pitch rate.

#### Roll rate: $\bar{\left|p\right|},{\left|p\right|}_{\text{max}}$

Both the maximum and mean absolute roll rates show that they are significantly reduced in the 5% case compared to control bees and not significantly in 1% and 2.5% groups. However, the overall comparison of control and exposed group (*G*_5_) shows reduction in both $\bar{\left|p\right|}\left(p=0.02,d=-0.97\right),{\left|p\right|}_{\text{max}}\left(p=0.03,d=-0.87\right)$. The increased proportion of hover and ascending sequences in the 5% concentration recordings may be a reason for the reduction in roll rate quantities, as these longitudinal motions involve small bank angles.

#### Body speeds

The mean body speeds are unaffected over the dataset in every comparison. ${\bar{\left|u\right|}}_{\text{max}}$ and ${\bar{\left|v\right|}}_{\text{max}}$ reduced in 2.5% to significantly affect the control vs exposed case. This trend is not continued in 5% case and cannot be conclusively linked to ethanol exposure.

### Wingstroke kinematic characteristics

Previous studies in near hover conditions [33, 34] found that the gross wingstroke is largely consistent, and small perturbations to the stroke are sufficient to carry out a variety of flying maneuvers. Wing motion characteristics under ethanol influence have not yet received such a study. Insect asynchronous flight muscles operate near mechanical resonance [35] and frequency deviations normally result in reduced performance. Hovering honey bees rely on relatively short amplitude and high frequency wingstrokes and maneuver via amplitude tuning [33, 36]. Insects may affect yaw axis acceleration with small changes in stroke plane angle and stroke amplitude [37]. In bulk stroke analysis, the 1% ethanol influence group showed a frequency increase (*p* ≪ 0.05, *d* = 1.04) and amplitude decrease (*p* ≪ 0.05, *d* = −0.13) relative to control. Conversely, a frequency decrease (*p* ≪ 0.05, *d* = −0.61 and *d* = −1.04) and amplitude increase (*p* ≪ 0.05, *d* = 0.94 and *d* = 1.47) were seen in 2.5% and 5% groups. The 1% group does not follow the trend of decreasing frequency with increased exposure level. In trial-wise analysis, the mean variables follow the same trend as the bulk analysis but the significance levels vary. The frequency increase in 1% group is significant (*p* < 0.01, *d* = 1.51), while the frequency decrease in all other groups is not. The amplitude increases remain significant in the 2.5% and 5% groups (*p* < 0.05, *d* = 1.03 and *d* = 1.17). An aerodynamic interpretation suggests that for an insect maintaining the same flight force, frequency decreases must be accompanied by amplitude increase [38]. The preliminary study [18] indicated that the frequency decreased for all groups (1%, 2.5%, 5%), and the 1% group did not increase as found in this study. This effect may be related to the previous study’s technical limitations: wingstroke frequency was determined by manually computing peak-to-peak periods on a small fraction of wingstrokes, averaging these periods per concentration group. The present study analyzed all of the wingstrokes (instead of a small subset) by applying a robust digitizing tool developed since [18]. Excluding the 1% concentration, the overall stroke amplitude increase with concentration is consistent across both methods.

In bulk wingstroke analysis, stroke plane inclination *β* increased in every ethanol-exposed group (1%, 2.5%, 5%) with a moderate effect size (*p* ≪ 0.05, *d* = 0.50, 0.74, 0.73). *β* as a control input affects both forward flight speed *u* and pitch rate *q* [39]. Although increasing *β* tends to increase forward speed, this effect can be mitigated by changes in flight force due to the frequency and amplitude changes. In trial-wise analysis, the increase is only significant in the 2.5% group (*p* < 0.05, *d* = 1.17).

In the bulk analysis, the dorsal bias *δ* decreased significantly in the 1% group (*p* ≪ 0.05, *d* = −0.59) but it increased significantly (*p* ≪ 0.05, *d* = 0.71) in 5% group and thus shows no clear trend. In trial-wise analysis, the changes are significant in 1% and 5% groups (*p* < 0.01, *d* = −1.60 and *p* < 0.05, *d* = 1.19).

A gradual mean loop size decrease with ethanol concentration (i.e., more planar wingstroke) is also visible in Fig 5 (top) with bulk analysis, while trial-wide analysis showed an increase in loop size at the 2.5% to 5% transition. The stroke amplitude increase is clearest at 5% ethanol, where the behavioral analysis shows a larger proportion of ascending sequences consistent with greater force production. The effect’s reduction in trial-wise analysis suggests the length of ascending periods may also contribute to the effect. The stroke history standard deviations are larger in the control group, an effect which is reduced in trial-wise analysis, consistent with the higher diversity of behavioral maneuvers in 0% flight. The mechanics and effect of non-planar wingstrokes are still not well understood [22, 40, 41] and require further aerodynamic analysis.

### Limitations and observations

Honey bees exposed to 10% ethanol solution did not initiate flight within 60 minutes in the test chamber, thus this study did not consider their flights. The 2.5% and 5% subjects initiated flight within 30 minutes of introduction to the test volume and displayed erratic ground movements prior to flight. Trials conducted at the 1% concentration in free-flight are quantitatively different from control insects and are distinct from the effects at higher concentrations, suggesting ethanol treatment effects are not a simple monotonic trend. For toxic pesticides, biphasic ‘hormesis’ effect has been observed [42, 43] in honeybees, where a small amount of exposure to a toxic element shows the opposite effect of the exposure to a bigger one. Honeybees treated with low dosage (1%) of ethanol were also difficult to distinguish behaviorally from the control group in our current and previous studies [18] and the distinction might be the result of hormesis, which needs further investigation.

Statistical analyses such as these are limited to quantifying effects and the relative likelihood of such a measurement occurring due to chance. They do not identify the physiological or neural mechanisms behind such effects. This analysis also does not account for the inter-dependence of variables. These are the first recorded quantitative high speed measurements of ethanol-exposed honey bee flight, and experimental limitations on the number of animals constrain the dataset size. Although previous literature has made most use of bulk wingstroke averages, varying trial lengths may leave bulk averages susceptible to bias towards longer trials. To account for this effect, we also analyzed the flight data set using a stereotypical wingstroke identified from the duration of each flight recording. This parallel analysis is not without limitation, as assigning one single frequency or amplitude to a whole flight trial does not capture the characteristics of individual wingstroke properties, and the methods must be interpreted by reference to each other. The trends of the differences persisted in both analyses, and were indicated as statistically significant when bulk wingstrokes were analyzed (versus trial-wise analysis), a combination which strengthens the study’s applicability.

## Conclusion

This paper presents the first quantitative high speed measurements of ethanol-exposed honey bee flight body and wing kinematic parameters in archival literature. Kinematic changes induced by exposure to ethanol concentrations from 0% to 5% were studied using behavioral analysis and statistical tools. The flight behavior is found to be turn dominated. The maximum heading and pitch rates reduce with increased ethanol exposure, while roll rates were affected at the 5% exposure level, with a potential link to changes in flight behavior. Wingstroke analysis indicates a frequency decrease and amplitude increase for 2.5% and greater ethanol concentration exposures. Wingstroke loop size decreased and wing inclination angle increased with increasing exposure level. Understanding the flight variables induced by this chemical manipulation in non-interacting flight conditions is an important result to distinguish the effects of chemically mediated social interactions with neighboring flyers from chemical effects themselves. The study of flight behavior of honey bees to ethanol exposure may also help us to better understand the mechanics of fine motor activity under the influence of ethanol.

## Supporting information

S1 File Raw datasets. Raw datasets of honey bee flights with different concentrations of ethanol exposure.
(ZIP)

S1 Video Ethanol exposed flight digitization. A sample video footage where a 0% bee and 1% bee are flying together. Their reconstructions and planar wing motion are shown.
(MP4)
